# Joint predictability of physical frailty/pre-frailty and subjective memory complaints on mortality risk among cognitively unimpaired older adults

**DOI:** 10.1007/s10433-023-00765-y

**Published:** 2023-05-18

**Authors:** Chia-Lin Li, Fiona F. Stanaway, Hsing-Yi Chang, Min-Chi Chen, Yu-Hsuan Tsai

**Affiliations:** 1 Department of Health Care Management, College of Management, Chang Gung University, 259 Wen-Hwa 1St Road, Kwei-Shan, Tao-Yuan, 33302 Taiwan; 2 Faculty of Medicine and Health, School of Public Health, University of Sydney, Sydney, NSW Australia; 3 Institute of Population Health Sciences, National Health Research Institutes, Maoli, Taiwan; 4 Department of Public Health, Biostatistics Consulting Center, College of Medicine, Chang Gung University, Taoyuan, Taiwan

**Keywords:** Frailty, Subjective memory complaints, Mortality, Taiwan

## Abstract

The aim of the present study was to investigate how frailty/pre-frailty in combination with subjective memory complaints predicts all-cause mortality in community dwelling cognitively unimpaired older adults. There were 1904 community-dwelling cognitively unimpaired persons aged 65 years or older who participated in the 2013 Taiwan National Health Interview Survey with a 5-year follow-up. Frailty was determined based on the fatigue, resistance, ambulation, illness, and loss of weight (FRAIL) scale. Two questions (“Do you have difficulties with your memory or attention?” and “Do you have difficulties with your memory only or attention only or both?”) were used to screen for subjective memory complaints (SMC). In this study, 11.9% of participants had both frailty/pre-frailty and SMC. A total of 239 deaths were recorded after 9009.5 person-years of follow-up. After adjustment for other factors, compared with participants who were physically robust with no SMC, participants who reported either SMC alone (HR = 0.88, 95% CI = 0.60–1.27) or were frail/pre-frail alone (HR = 1.32, 95% CI = 0.90–1.92) had no significantly increased mortality risk. However, coexisting frailty/pre-frailty and SMC was associated with a significantly increased hazard ratio for mortality of 1.48 (95% CI = [1.02–2.16]). Our results highlight the high prevalence of co-occurring frailty/pre-frailty and SMC and that this co-occurrence is associated with an increased risk of mortality among cognitively unimpaired older adults.

## Introduction

Subjective memory complaints (SMC) is a condition characterized by self-perceived memory deficits in everyday life. Most previous studies of factors associated with SMC in older adults have focused on normal aging processes, poorer objective memory performance, self-perceived health, and the presence of psychiatric and neurologic disorders (Elfgren et al. 2010; Genziani et al. 2013; Lehrner et al. 2014; Montejo et al. 2014; Montejo Carrasco et al. 2017). SMC has been linked to an increased risk of falls, health care utilization, nursing home admission, and incident dementia (Waldorff et al. 2009; Mitchell et al. 2014; Al-Sari et al. 2017). Therefore, SMC could have value as a clinical marker that could be used to identify individuals who require more care.

Frailty is an important concept as it can enable the identification of older individuals with increased vulnerability who are at higher risk of adverse outcomes. Physical frailty is also associated with increased mortality (Lahousse et al. 2014). However, among cognitively unimpaired older persons, associations between physical frailty and mortality reported in the literature have been inconsistent (Avila-Funes et al. 2009; Feng et al. 2017). Feng et al. (2017) analyzed data from 2375 community-living Singaporeans aged 55 years and older participating in the Singapore Longitudinal Ageing Study. They found that the presence of frailty or pre-frailty in older persons without cognitive impairment at baseline was not associated with an increased risk of mortality during an approximately 3-year period.

Robertson et al. (2014) conducted a cross-sectional study using data from The Irish Longitudinal Study on Aging that included participants aged 50 and older without a history of stroke, Parkinson’s disease, severe cognitive impairment, or antidepressant use. The authors found worse performance across multiple cognitive domains such as memory, processing speed, executive function, and attention in those who were pre-frail or frail. Wu et al. (2015) also conducted a cross-sectional study using data from the I-Lan Longitudinal Aging Study including a total of 1839 community residents aged 50 years or older without dementia or global cognitive impairment or cognitive complaints at baseline. They found that frail individuals were 2.55 times more likely to have memory-domain impairment than robust individuals. These findings highlight that SMC may be under-recognized among community dwelling cognitively unimpaired older people and that a vulnerable subgroup of these older adults will have both frailty/pre-frailty and SMC. Despite this evidence, data about the epidemiology of co-occurring frailty/pre-frailty and SMC among cognitively unimpaired older persons have not been well characterized. Little is known about how frailty/pre-frailty in combination with SMC is associated with mortality risk among cognitively unimpaired older persons.

Previous studies show that older adults with pre-frailty have different profiles in terms of clinical, functional, and behavioral and biomarker characteristics to robust individuals (Abellan van Kan et al. 2008a). It has also been reported that older adults with pre-frailty and frailty share similarities in pathological aging (Ruan et al. 2015). There is increasing evidence that prompt interventions in older adults with pre-frailty to achieve a better outcome than intervening at a later stage (Abellan van Kan et al. 2008a). Therefore, in this study, we have merged the categories of pre-frailty and frailty as a single category.

In view of these considerations, we have analyzed data from a 5-year prospective study of a national sample of community-dwelling cognitively unimpaired older adults in Taiwan. The aims of the present study were twofold. First, we aimed to describe the distribution of co-occurring frailty/pre-frailty and SMC among cognitively unimpaired older people. Second, we aimed to examine the association between coexisting frailty/pre-frailty and SMC and mortality in cognitively unimpaired older adults. We hypothesized that those with coexisting frailty/pre-frailty and SMC would have an increased risk of all-cause mortality.

## Methods

### Study population

This was a prospective study involving participants in the National Health Interview Survey (NHIS) in Taiwan, 2013. The sample design of the NHIS has been described in detail previously (Li et al. 2020; Li et al. 2021a, b). All study participants provided signed written informed consent. There were a total of 3203 individuals aged 65 years and older of whom we excluded 172 persons with a pre-existing dementia or Parkinson's disease diagnosis and 461 persons with incomplete data for the Mini-Mental State Examination (MMSE), physical frailty, depressive symptoms, or SMC. Out of these 2570 participants, we excluded a further 666 participants who were diagnosed as cognitively impaired (MMSE score < 18 for participants who were illiterate and had no schooling, < 21 for those with 1–6 years of education, and < 25 for those with 7 or more years of education) (Folstein et al. 1975; Katzman et al. 1988). This resulted in 1904 eligible participants for the analysis. The study cohort was followed until death or the end of the study period (December 31, 2018). Deaths were confirmed by the computerized data files of the National Register of Deaths. The processes of data set linkage and statistical analysis were performed at the Data Science Center of the Ministry of Health and Welfare. This study was approved by the relevant institutional review board.

### Measures

#### Assessment of physical frailty and SMC

In this study, frailty and pre-frailty were determined based on the fatigue, resistance, ambulation, illness, and loss of weight (FRAIL) scale (Abellan van Kan et al. 2008a, b). Fatigue was assessed by asking individuals how much of the time they felt tired in the past 1 week. Responses of “all” or “most of the time” were given a score of 1. Resistance was assessed by asking individuals whether they have difficulty climbing ten steps, and ambulation was assessed by asking individuals whether they have difficulty walking 100 m (about one block). Responses of “some difficulty”, “much difficulty”, or “unable to carry out” received a score of 1. Illnesses were assessed by asking individuals if a medical professional has ever told them that they have any of the following conditions: diabetes, heart disease, hypertension, stroke, asthma, kidney disease, chronic lung disease, cancer, and arthritis. A report of five or more illnesses received a score of 1. Loss of weight was assessed by body mass index (BMI was calculated as weight [kg] divided by height squared [m^2^]), and participants scored 1 point if their BMI was less than 18.5 kg/m^2^ (Woo et al. 2012). FRAIL scores range from 0 to 5. Participants with scores ranging from 3 to 5 were defined as frail, 1–2 as pre-frail, and 0 as robust. We grouped frail and pre-frail participants into a single category.

SMC was assessed in the self-report questionnaire by two questions. The first question was “Do you have difficulties with your memory or attention?”. The response categories were “no difficulty”, “some difficulty”, “much difficulty”, or “completely unable”. Participants responding with “some difficulty”, “much difficulty”, or “completely unable” were asked a second question, “Do you have difficulties with your memory only or attention only or both?”. The response categories were “memory alone”, “attention alone”, or “both memory and attention”. SMC was defined as reporting some difficulty, much difficulty or being completely unable to use their memory alone or both their memory and attention. The same approach to screen for SMC in this study has been shown to be predictive of incident dementia in our previous study of older adults (Li et al. 2021a).

#### Assessment of baseline characteristics

Depressive symptoms were assessed by the 10 item version of the Center for Epidemiologic Studies Depression Scale (CES-D) (Radloff 1977; Andresen et al. 1994). Participants with scores ranging from 10 to 30 were defined as having depressive symptoms. Demographic and health information such as age, sex, years of education, marital status (married or living with partner; yes/no), smoking status (current or former smoker; yes/no) and physical activity (individuals were classified as active if they did report engaging in any kind of leisure activity during the last one month; yes/no) were obtained from the questionnaires. Individuals were categorized as having fallen if they self-reported at least one fall during the previous year. Hospital admissions and emergency department visits were assessed by respondents’ self-reporting at least one hospital admission or emergency department visit in the previous year before the interview (any or none).

### Statistical analysis

We used Pearson’s Chi-square test to compare baseline characteristics between surviving and non-surviving participants. We also used Pearson’s Chi-square test to compare baseline characteristics between participants with frailty/pre-frailty and/or SMC. We also used Pearson’s Chi-square test to compare baseline characteristics between included and excluded participants. Cox proportional-hazards models were used to investigate the association between baseline characteristics and all-cause mortality. The proportional hazards assumption (tested using Schoenfeld residuals) was not violated. Hazard ratios (HRs) and 95% confidence intervals (95% CIs) for mortality were estimated. We used Cox regression models to examine the joint associations between frailty/pre-frailty, SMC and mortality. All analyses were conducted using SAS statistical software, version 9.4 (SAS Institute, Cary, NC).

## Results

Table 1 compares baseline characteristics between surviving and non-surviving participants. Surviving participants were significantly younger and were more likely to be female, have higher education levels, be married or living with partner, and likely to be physically active. They were less likely to be current or former smokers, have depressive symptoms, be hospitalized or visited the emergency department during the past year. Surviving participants were also less likely to be frail, to report SMC, or to have coexisting frailty/pre-frailty and SMC.

**Table 1:** Baseline characteristics of participants

	Total sample (*N* = 1904)	Survivors (*N* = 1665)	Deceased (*N* = 239)	*P*-value^*^
Age (%)				< 0.0001
65–74 years	1201 (63.1)	1112 (66.8)	89 (37.2)	
75 + years	703 (36.9)	553 (33.2)	150 (62.8)	
Sex (% female)	958 (50.3)	874 (52.5)	84 (35.2)	< 0.0001
Education (%)				0.0068
0 years	511 (26.8)	440 (26.4)	71 (29.7)	
1–6 years	890 (46.7)	765 (46.0)	125 (52.3)	
7 + years	503 (26.4)	460 (27.6)	43 (18.0)	
Marital status (% married or living with partner)	1285 (67.5)	1148 (69.0)	137 (57.3)	0.0003
Current or former smoker (% yes)	532 (27.9)	439 (26.4)	93 (38.9)	< 0.0001
Physically active (% yes)	1044 (55.0)	945 (56.8)	99 (41.8)	< 0.0001
Depressive symptoms (% yes)	196 (10.3)	150 (9.0)	46 (19.3)	< 0.0001
Fallen during past year (% yes)	263 (13.8)	222 (13.3)	41 (17.2)	0.1102
Hospitalization (% yes)	226 (11.9)	176 (10.6)	50 (20.9)	< 0.0001
Emergency department visit (% yes)	302 (15.9)	249 (15.0)	53 (22.2)	0.0043
Frailty status (%)				< 0.0001
Robust	1430 (75.1)	1290 (77.5)	140 (58.6)	
Pre-Frail	420 (22.1)	344 (20.7)	76 (31.8)	
Frail	54 (2.8)	31 (1.9)	23 (9.6)	
SMC (% yes)	641 (33.7)	548 (32.9)	93 (38.9)	0.0665
Frail/Pre-frail and/or SMC (%)				< 0.0001
No/No	1015 (53.3)	915 (55.0)	100 (41.8)	
No/Yes	415 (21.8)	375 (22.5)	40 (16.7)	
Yes/No	248 (13.0)	202 (12.1)	46 (19.3)	
Yes/Yes	226 (11.9)	173 (10.4)	53 (22.2)	

^*^Categorical variables were compared using Pearson’s Chi-square test and shown as percentages

Table 2 presents the baseline characteristics of participants by physical frailty/pre-frailty and/or SMC. The prevalence of co-occurring frailty/pre-frailty with SMC was 11.9%. Compared to participants who were physical robust without SMC, participants with co-occurring frailty/pre-frailty and SMC were less likely to be married or living with a partner, be a current or former smoker, and be physically active. They were more likely to be older, female, have less education, report depressive symptoms, have fallen during the last year, and have been hospitalized or visited the emergency department during the past year.

**Table 2:** Baseline characteristics of participants by the presence of physical frailty/pre-frailty and/or subjective memory complaints

Variables	Frail or pre-frail/subjective memory complaints				
	No/No (*N* = 1015)	No/Yes (*N* = 415)	Yes/No (*N* = 248)	Yes/Yes (*N* = 226)	*P*-value^*^
Age (%)					< 0.0001
65–74	729 (71.8)	260 (62.7)	118 (47.6)	94 (41.6)	
75 +	286 (28.2)	155 (37.4)	130 (52.4)	132 (58.4)	
Sex (% female)	449 (44.2)	214 (51.6)	159 (64.1)	136 (60.2)	< 0.0001
Education (%)					< 0.0001
0 years	205 (20.2)	110 (26.5)	104 (41.9)	92 (40.7)	
1–6 years	477 (47.0)	213 (51.3)	106 (42.7)	94 (41.6)	
7 + years	333 (32.8)	92 (22.2)	38 (15.3)	40 (17.7)	
Marital status (% married or living with partner)	744 (73.3)	280 (67.5)	138 (55.7)	123 (54.4)	< 0.0001
Current or former smoker (% yes)	314 (30.9)	109 (26.3)	57 (23.0)	52 (23.0)	0.0125
Physically active (% yes)	619 (61.1)	252 (60.7)	90 (36.4)	83 (37.1)	< 0.0001
Depressive symptoms (% yes)	43 (4.2)	29 (7.0)	58 (23.4)	66 (29.2)	< 0.0001
Fallen during past year (% yes)	109 (10.8)	57 (13.7)	48 (19.4)	49 (21.7)	< 0.0001
Hospitalization (% yes)	77 (7.6)	41 (9.9)	48 (19.4)	60 (26.6)	< 0.0001
Emergency department visit (% yes)	116 (11.4)	65 (15.7)	60 (24.2)	61 (27.0)	< 0.0001

^*^Categorical variables were compared using Pearson’s Chi-square test and shown as percentages

Table 3 presents the number of deaths, person-years, mortality rate, and crude and adjusted HR and 95% CIs. The crude mortality rate in the whole sample was 26.5 per 1,000 person-years. After adjustment for age, sex, education, marital status, smoking status, physical activity, and depressive symptoms, the adjusted HR for mortality was 0.89 (95% CI = [0.62–1.29]) in those with SMC alone, 1.40 (95% CI = [0.96–2.04]) in those with frailty/pre-frailty alone, and 1.66 (95% CI = [1.15–2.39]) in those with coexisting frailty/pre-frailty and SMC, compared to robust individuals without SMC. After adjustment for age, sex, education, marital status, smoking status, physical activity, depressive symptoms, history of falls, hospitalization, and emergency department visits during the past year, the adjusted HR for mortality was 0.88 (95% CI = [0.60–1.27]) in those with SMC alone, 1.32 (95% CI = [0.90–1.92]) in those with frailty/pre-frailty alone, and 1.48 (95% CI = [1.02–2.16]) in those with coexisting frailty/pre-frailty and SMC, compared to robust individuals without SMC.

**Table 3:** Crude and adjusted hazard rations (HR) and 95% confidence intervals (95% CI) for incidence of mortality

	Overall	Robust		Frail/Pre-Frail	
		Without SMC	With SMC	Without SMC	With SMC
*N*	1904	1015	415	248	226
Deaths	239	100	40	46	53
Person years	9009.5	4856.6	1991.6	1135.5	1025.8
Incidence Rate (per 1000 person-years)	26.5	20.6	20.1	40.5	51.7
Model 1					
HR (95% CI)		Reference	0.98 (0.68–1.41)	1.99 (1.40–2.82)	2.54 (1.82–3.55)
* P*-value			0.8967	0.0001	< 0.0001
Model 2					
HR (95%CI)		Reference	0.89 (0.62–1.29)	1.40 (0.96–2.04)	1.66 (1.15–2.39)
* P*-value			0.5409	0.0800	0.0065
Model 3					
Adjusted HR (95% CI)		Reference	0.88 (0.60–1.27)	1.32 (0.90–1.92)	1.48 (1.02–2.16)
* P*-value			0.4776	0.1530	0.0397

Model 2: adjusted for age, sex, education, marital status, current or former smoker, physically active, and depressive symptoms

Model 3: adjusted for age, sex, education, marital status, current or former smoker, physically active, depressive symptoms, have fallen, hospitalization, and emergency department visits during the past year

Table 4 presents the comparison of baseline characteristics between those who were included and excluded from this study. Study participants were significantly younger, had higher education levels, were more likely to be married or living with a partner, and more likely to be physically active. They were less likely to have a history of falls, hospitalization, or an emergency department visit during the past year.

**Table 4:** Characteristics of included versus excluded participants

	Included (*N* = 1904)	Excluded (*N* = 461)	*P*-value^*^
Age (%)			< 0.0001
65–74 years	1201 (63.1)	174 (37.7)	
≥ 75 years	703 (36.9)	287 (62.3)	
Sex (% female)	958 (50.3)	224 (48.6)	0.5063
Education (%)			< 0.0001
0 years	511 (26.8)	168 (38.7)	
1–6 years	890 (46.7)	188 (43.3)	
7 + years	503 (26.4)	78 (18.0)	
Marital status (% married or living with partner)	1285 (67.5)	245 (53.3)	< 0.0001
Current or former smoker (% yes)	532 (27.9)	134 (29.2)	0.5923
Physically active (% yes)	1044 (55.0)	148 (40.8)	< 0.0001
Fallen during past year (% yes)	263 (13.8)	96 (20.9)	0.0001
Hospitalization (% yes)	226 (11.9)	104 (30.9)	< 0.0001
Emergency department visit (% yes)	302 (15.9)	109 (32.7)	< 0.0001

^*^Categorical variables were compared using Pearson’s Chi-square test and shown as percentages

## Discussion

Our study found that 11.9% of participants had co-occurring frailty/pre-frailty and SMC. Our results confirm our hypothesis that the co-occurrence of frailty/pre-frailty and SMC among cognitively unimpaired older adults is significantly associated with an increased risk of all-cause mortality. Moreover, compared with participants who were physically robust without SMC, participants who had either frailty/pre-frailty alone or had SMC alone did not have an increased risk of mortality. These findings highlight that among community dwelling cognitively unimpaired older adults, the concurrent presence of frailty/pre-frailty and SMC has incremental predictive validity for all-cause mortality.

Our results contribute to the literature by providing new data on co-occurring frailty/pre-frailty and SMC associated with increased mortality in a population-based national sample of cognitively unimpaired older adults. In this study, after adjustment for other factors, compared to physically robust participants without SMC, frail/pre-frail participants with SMC had an HR for all-cause mortality of 1.48 (95% CI = [1.02–2.16]). These estimates are similar to those from the Italian Longitudinal Study on Aging (Solfrizzi et al. 2017). The authors assessed subjective cognitive decline (SCD) with the question “Do you feel you have more problems with memory than most?” and adjusted for the Geriatric Depression Scale (GDS)-30 total score. They reported that participants with both frailty/pre-frailty and pre-mild cognitive impairment (SCD) had a HR for all-cause mortality of 1.74 (95% CI = [1.07–2.83]) over 3.5 years and 1.39 (95% CI = [1.03–2.00]) over 7-years of follow-up.

We failed to find an association between frailty/pre-frailty alone and all-cause mortality, although frail/pre-frail participants who had SMC did have an increased risk of mortality. There are several possible explanations for this finding. It could be that frail/pre-frail participants with memory complaints have greater vulnerability which manifests itself as increased mortality compared to those participants with frail/pre-frail alone. Physical frailty/pre-frailty and SMC may have common risk factors, such as poorer objective memory performance, slower gait speed, worse cognitive performance, and depressive symptoms (Elfgren et al. 2010; Genziani et al. 2013; Lehrner et al. 2014; Wu et al. 2015; Lin et al. 2022; Montejo Carrasco et al. 2017; Chang et al. 2019). Lin et al. (2022) performed an investigation of adults aged 60 years or older with memory complaints recruited from an outpatient geriatric service in Brazil and found that pre-frailty was associated with poor performance in the memory domain. Moreover, they found that slower gait speed was associated with a worse performance in the memory domain. Slow gait speed has been linked to risk of mortality (Studenski et al. 2011). Our previous longitudinal study found that among community dwelling cognitively unimpaired older adults aged 65 or older, frail/pre-frail persons with SMC were at increased risk of incident dementia (Li et al. 2021a). Maxwell and colleagues performed a retrospective cohort study of long-stay home care of clients aged 50 or older. They found that frail or pre-frail participants with dementia had increased mortality (Maxwell et al. 2019). Our data showed that frail/pre-frail participants with SMC more likely to have depressive symptoms. Chang and colleagues analyzed data of 3352 individuals aged 60 or older who participated in the Taiwan Longitudinal Study of Aging from 1989 to 2007 and concluded that depressive symptoms in frail older adults were associated with a lower likelihood of reversal of frailty and increased mortality risk (Chang et al. 2019). These observations provide possible biology pathway behind our finding that frailty/pre-frailty in combination with SMC increases mortality risk.

We did not find an association between SMC alone and all-cause mortality among cognitively unimpaired older adults. It is possible that better health status is contributing to the association between physical robust and no excess mortality in older adults with memory complaints. Similar results have been reported by Siersma and colleagues from a prospective cohort study of 758 patients aged 65 years and older visiting their general practitioner with four years of follow-up (Siersma et al. 2013). The authors defined SMC according to the response to “How would you judge your memory?”. There were five response categories: “less good”, “poor”, or “miserable” were classified as SMC, while participants rating their memory as “excellent” or “good” were classified as no SMC. After adjustment for cognitive impairment and other factors, they found that the presence of SMC was not significantly associated with an increased risk of all-cause mortality.

There are some limitations in our study that should be noted. Our analytic sample could be biased due to included participants being limited to those who were able to complete the assessments for cognitive function, physical frailty/pre-frailty, depressive symptoms, and SMC. The comparison of baseline characteristics between respondents who were included (*N* = 1904) and excluded (*N* = 461) from this study (Table 4) suggests that our sample could be biased toward individuals of younger age, with a higher education level, who are married or living with partners, physical active, and who were less likely to have a history of falls, hospitalization, or emergency department visit in the year before the interview. It suggests that our sample could be biased toward individuals with better health status. Thus, the observed crude mortality rate of 26.5 per 1,000 person-years may be an underestimate and the association between frailty/pre-frailty and SMC with mortality may also be underestimated. Moreover, history of falls and healthcare utilization can occur as a result of frailty, but that they can also lead to frailty. Given that the sequence of events is not clear from the data collected, we have provided the analysis results with and without adjustment for these variables (Table 3, Model 2 and Model 3). The results do not actually change a lot, although without adjustment for these variables, frailty/pre-frailty alone showed a marginally significant association with mortality (Table 3, Model 2). We consider that the effect of frailty/pre-frailty alone on mortality could be clinically important, not only because of the adjusted point estimate of 1.32 (Table 3, Model 3), but also because the confidence interval was almost entirely above 1. It is likely that lack of sufficient statistical power resulted in the failure to reach statistical significance. The frailty/pre-frailty condition we assessed by the FRAIL scale was based on self-report, and therefore, recall bias was unavoidable. However, the strengths of using the FRAIL scale are relatively simple and easy to use. As this study was an observational study and frailty and SMC were assessed at the same time, there are limitations on the causal interpretation of our study findings. Further investigation is needed to explore the underlying cause of mortality in people with frail/pre-frail and SMC.

## Conclusions

We found that 2.8%, 22.1% and 33.7% of Taiwanese cognitively unimpaired adults aged 65 years and above were frail, pre-frail, or had SMC, respectively. The prevalence of SMC is consistent with most other studies where the prevalence of SMC in older adults lies within the range of 25% to 50% (Jonker et al. 2000; Montejo et al. 2011). It is notable that as many as 51.9% of frail participants and 47.1% of pre-frail participants had SMC. Moreover, our findings suggest that the concurrent presence of frailty/pre-frailty and SMC has incremental predictive validity for older adults without cognitive impairment who are at increased risk of mortality in the absence of laboratory and clinical indicators. We suggest that clinicians should pay careful attention to memory complaints in their frail/pre-frail older patients at regular clinic attendances to identify individuals who would benefit most from interventions aimed at preventing death. In summary, our results indicate that a substantial proportion of frail/pre-frail older adults have memory complaints. Our findings highlight the importance of screening for SMC to identify frail/pre-frail persons who may need interventions aimed at improving survival among cognitively unimpaired older adults.

## References

[CR1] Abellan van Kan G, Rolland Y, Bergman H, Morley JE, Kritchevsky SB, Vellas B (2008). The I.A.N.A Task Force on frailty assessment of older people in clinical practice. J Nutr Health Aging.

[CR2] Abellan van Kan G, Rolland YM, Morley JE, Vellas B (2008). Frailty: toward a clinical definition. J Am Med Dir Assoc.

[CR3] Al-Sari UA, Tobias JH, Archer H, Clark EM (2017). Do subjective memory complaints predict falls, fractures and healthcare utilization? A two-year prospective study based on a cohort of older women recruited from primary care. Int J Geriatr Psychiatry.

[CR4] Andresen EM, Malmgren JA, Carter WB, Patrick DL (1994). Screening for depression in well older adults: evaluation of a short form of the CES-D (Center for Epidemiologic Studies Depression Scale). Am J Prev Med.

[CR5] Ávila-Funes JA, Amieva H, Barberger-Gateau P, Le Goff M, Raoux N, Ritchie K, Carrière I, Tavernier B, Tzourio C, Gutiérrez-Robledo LM, Dartigues JF (2009). Cognitive impairment improves the predictive validity of the phenotype of frailty for adverse health outcomes: the three-city study. J Am Geriatr Soc.

[CR6] Chang HY, Fang HL, Ting TT, Liang J, Chuang SY, Hsu CC, Wu CY, Pan WH (2019). The co-occurrence of frailty (accumulation of functional deficits) and depressive symptoms, and its effect on mortality in older adults: a longitudinal study. Clin Interv Aging.

[CR7] Elfgren C, Gustafson L, Vestberg S, Passant U (2010). Subjective memory complaints, neuropsychological performance and psychiatric variables in memory clinic attendees: a 3-year follow-up study. Arch Gerontol Geriatr.

[CR8] Feng L, Zin Nyunt MS, Gao Q, Feng L, Yap KB, Ng TP (2017). Cognitive frailty and adverse health outcomes: findings from the Singapore longitudinal ageing studies (SLAS). J Am Med Dir Assoc.

[CR9] Folstein MF, Folstein SE, McHugh PR (1975). Mini-mental state. A practical method for grading the cognitive state of patients for the clinician. J Psychiatr Res.

[CR10] Genziani M, Stewart R, Béjot Y, Amieva H, Artero S, Ritchie K (2013). Subjective memory impairment, objective cognitive functioning and social activity in French older people: findings from the Three Cities study. Geriatr Gerontol Int.

[CR11] Jonker C, Geerlings MI, Schmand B (2000). Are memory complaints predictive for dementia? A review of clinical and population-based studies. Int J Geriatr Psychiatry.

[CR12] Katzman R, Zhang MY, Qu OY, Wang ZY, Liu WT, Yu E, Wong SC, Salmon DP, Grant I (1988). A Chinese version of the Mini-Mental State Examination; impact of illiteracy in a Shanghai dementia survey. J Clin Epidemiol.

[CR13] Lahousse L, Maes B, Ziere G, Loth DW, Verlinden VJ, Zillikens MC, Uitterlinden AG, Rivadeneira F, Tiemeier H, Franco OH, Ikram MA, Hofman A, Brusselle GG, Stricker BH (2014). Adverse outcomes of frailty in the elderly: the Rotterdam Study. Eur J Epidemiol.

[CR14] Lehrner J, Moser D, Klug S, Gleiß A, Auff E, Dal-Bianco P, Pusswald G (2014). Subjective memory complaints, depressive symptoms and cognition in patients attending a memory outpatient clinic. Int Psychogeriatr.

[CR15] Li CL, Chang HY, Shyu YL, Stanaway FF (2021). Relative role of physical frailty and poor cognitive performance in progression to dementia. J Am Med Dir Assoc.

[CR16] Li CL, Chang HY, Stanaway FF (2020). Combined effects of frailty status and cognitive impairment on health-related quality of life among community dwelling older adults. Arch Gerontol Geriatr.

[CR17] Li CL, Chiu YC, Shyu YL, Stanaway FF, Chang HY, Bai YB (2021). Does physical activity protect older persons with frailty and cognitive impairment from excess all-cause mortality?. Arch Gerontol Geriatr.

[CR18] Lin SM, Apolinário D, Vieira Gomes GC, Cassales Tosi F, Magaldi RM, Busse AL, Gil G, Ribeiro E, Satomi E, Aprahamian I, Filho WJ, Suemoto CK (2022). Association of cognitive performance with frailty in older individuals with cognitive complaints. J Nutr Health Aging.

[CR19] Maxwell CJ, Mondor L, Hogan DB, Campitelli MA, Bronskill SE, Seitz DP, Wodchis WP (2019). Joint impact of dementia and frailty on healthcare utilisation and outcomes: a retrospective cohort study of long-stay home care recipients. BMJ Open.

[CR20] Mitchell AJ, Beaumont H, Ferguson D, Yadegarfar M, Stubbs B (2014). Risk of dementia and mild cognitive impairment in older people with subjective memory complaints: meta-analysis. Acta Psychiatr Scand.

[CR21] Montejo P, Montenegro M, Fernandez MA, Maestu F (2011). Subjective memory complaints in the elderly: Prevalence and influence of temporal orientation, depression and quality of life in a population-based study in the city of Madrid. Aging Ment Health.

[CR22] Montejo P, Montenegro M, Fernández-Blázquez MA, Turrero-Nogués A, Yubero R, Huertas E, Maestú F (2014). Association of perceived health and depression with older adults' subjective memory complaints: contrasting a specific questionnaire with general complaints questions. Eur J Ageing.

[CR23] Montejo Carrasco P, Montenegro-Peña M, López-Higes R, Estrada E, Prada Crespo D, Montejo Rubio C, García Azorín D (2017). Subjective memory complaints in healthy older adults: fewer complaints associated with depression and perceived health, more complaints also associated with lower memory performance. Arch Gerontol Geriatr.

[CR24] Ruan Q, Yu Z, Chen M, Bao Z, Li J, He W (2015). Cognitive frailty, a novel target for the prevention of elderly dependency. Ageing Res Rev.

[CR25] Radloff LS (1977). The CES-D scale: a self-report depression scale for research in the general population. Appl Psychol Meas.

[CR26] Robertson DA, Savva GM, Coen RF, Kenny RA (2014). Cognitive function in the prefrailty and frailty syndrome. J Am Geriatr Soc.

[CR27] Siersma V, Waldemar G, Waldorff FB (2013). Subjective memory complaints in primary care patients and death from all causes: a four-year follow-up. Scand J Prim Health Care.

[CR28] Solfrizzi V, Scafato E, Seripa D, Lozupone M, Imbimbo BP, D'Amato A, Tortelli R, Schilardi A, Galluzzo L, Gandin C, Baldereschi M, Di Carlo A, Inzitari D, Daniele A, Sabbà C, Logroscino G, Panza F (2017). Reversible cognitive frailty, Dementia, and all-cause mortality. The Italian Longitudinal Study on Aging. J Am Med Dir Assoc.

[CR29] Studenski S, Perera S, Patel K, Rosano C, Faulkner K, Inzitari M, Brach J, Chandler J, Cawthon P, Connor EB, Nevitt M, Visser M, Kritchevsky S, Badinelli S, Harris T, Newman AB, Cauley J, Ferrucci L, Guralnik J (2011). Gait speed and survival in older adults. JAMA.

[CR30] Waldorff FB, Siersma V, Waldemar G (2009). Association between subjective memory complaints and nursing home placement: a four-year follow-up. Int J Geriatr Psychiatry.

[CR31] Woo J, Leung J, Morley JE (2012). Comparison of frailty indicators based on clinical phenotype and the multiple deficit approach in predicting mortality and physical limitation. J Am Geriatr Soc.

[CR32] Wu YH, Liu LK, Chen WT, Lee WJ, Peng LN, Wang PN, Chen LK (2015). Cognitive function in individuals with physical frailty but without Dementia or cognitive complaints: results from the I-Lan longitudinal aging study. J Am Med Dir Assoc.

